# A pilot study of ezetimibe *vs.* atorvastatin for improving peripheral microvascular endothelial function in stable patients with type 2 diabetes mellitus

**DOI:** 10.1186/s12944-015-0028-z

**Published:** 2015-04-23

**Authors:** Seigo Sugiyama, Hideaki Jinnouchi, Kunio Hieshima, Noboru Kurinami, Tomoko Suzuki, Fumio Miyamoto, Keizo Kajiwara, Kunihiko Matsui, Tomio Jinnouchi

**Affiliations:** Diabetes Care Center, Jinnouchi Hospital, 6-2-3 Kuhonji, Chuo-ku, Kumamoto, 862-0976 Japan; Diabetes Care Center, Cardiovascular Division, Jinnouchi Hospital, 6-2-3 Kuhonji, Chuo-ku, Kumamoto, 862-0976 Japan; Department of Cardiovascular Medicine, Faculty of Life Sciences, Graduate School of Medical Science, Kumamoto University, 1-1-1 Honjo, Chuo-ku, Kumamoto, 862-8556 Japan; Division of Preventive Cardiology, Department of Cardiovascular Medicine, Kumamoto University Hospital, 1-1-1 Honjo, Chuo-ku, Kumamoto, 862-8556 Japan; Department of Community Medicine, Kumamoto University Hospital, 1-1-1 Honjo, Chuo-ku, Kumamoto, 862-8556 Japan

**Keywords:** Diabetes, Ezetimibe, Statins, Cholesterol absorption, Endothelial function, Microvasculature, Free fatty acid

## Abstract

**Background:**

Elevated cholesterol in type 2 diabetes mellitus (DM) can cause endothelial dysfunction. An effective clinical therapy to improve endothelial dysfunction remains to be established. Different cardiovascular actions between treatments for the inhibition of cholesterol absorption and the suppression of cholesterol synthesis for achieving improvement in endothelial function are unknown in DM.

**Methods:**

Stable patients with type 2 DM and mildly elevated low-density lipoprotein cholesterol were enrolled. We evaluated peripheral microvascular endothelial function using reactive hyperemia peripheral arterial tonometry (RH-PAT) examination and calculated a natural logarithmic transformed value for the RH-PAT index (LnRHI). We randomly assigned 33 patients to each monotherapy: cholesterol synthesis suppression using atorvastatin (5 mg/day, *n* = 16) or cholesterol absorption inhibition using ezetimibe (10 mg/day, *n* = 17). Patients were prospectively followed for 6 months. Serum lipids and LnRHI were repeatedly examined before and after each therapy.

**Results:**

LDL significantly decreased in both groups, but the percent changes of LDL showed a greater decrease in the atorvastatin group compared with the ezetimibe group (−34.5 ± 7.8% *vs.* −21.9 ± 9.6%, p < 0.01). Serum levels of non-esterified free fatty acids (NEFA) significantly decreased in the ezetimibe group but not in the atorvastatin group (ezetimibe group: 561.1 ± 236.8 to 429.7 ± 195.9, p < 0.01; atorvastatin group: 538.8 ± 319.5 to 520.2 ± 227.3, p = 0.75). The percent decrease in NEFA was significantly greater in the ezetimibe group compared with the atorvastatin group (−19.9 ± 27.4% *vs*. 11.3 ± 44.1%, p < 0.05). LnRHI showed a significant increase in the ezetimibe group but not in the atorvastatin group (ezetimibe group: 0.471 ± 0.157 to 0.678 ± 0.187, p < 0.01; atorvastatin group: 0.552 ± 0.084 to 0.558 ± 0.202, p = 0.64). The percent changes in LnRHI were significantly greater in the ezetimibe group compared with the atorvastatin group (63.3 ± 89.2% *vs*. 7.4 ± 41.2%, p < 0.05).

**Conclusions:**

In patients with type 2 DM, ezetimibe monotherapy significantly reduced LDL and NEFA, and improved peripheral microvascular endothelial dysfunction. Ezetimibe could potentially exhibit beneficial effects on lipid disorders and microvascular endothelial dysfunction in DM.

## Background

Type 2 diabetes mellitus (DM) is a high-risk clinical condition for cardiovascular disease [1]. Treatment strategies that may exhibit favorable effects on cardiovascular function are thought to have additionally beneficial clinical value in DM. Vascular endothelial dysfunction is a significant and independent predictor of future cardiovascular events [2], and the presence of DM and elevated low-density lipoprotein cholesterol (LDL) are recognized as important pathological conditions leading to endothelial dysfunction in clinical practice [3]. In patients with endothelial dysfunction, it has been reported that improvement in endothelial function with optimal medical treatments successfully improved cardiovascular prognosis in patients with coronary artery disease [4]; however, an effective treatment strategy to improve endothelial dysfunction is still uncertain in DM [3,5]. Increased attention has been focused on investigating and developing practical strategies for improving vascular endothelial function clinically [3]. Reactive hyperemia peripheral arterial tonometry (RH-PAT) is a simple, easy, non-invasive, and reproducible physiological examination for testing peripheral microvascular endothelial function in the fingertip [6,7]. We previously reported the clinical utility and significance of RH-PAT testing in terms of prognostic value [8,9], and on the interventional effects in patients with risk factors for cardiovascular disease [10,11].

LDL-lowering therapy is a promising and effective strategy for preventing atherothrombotic events in high-risk patients, and has been established as effective in DM [12]. There are two strategies to reduce LDL in clinical practice [1]: (1) suppression of endogenous cholesterol synthesis in the liver by statins; and (2) inhibition of cholesterol absorption in the intestine through blocking Niemann-Pick C1-Like 1 (NPC1L1) using ezetimibe [2]. Statins are generally better at lowering LDL than ezetimibe, but the clinical difference of both on cardiovascular function is not well investigated in DM.

The benefits of statins for patients with DM have been established in large-scale clinical trials [3]. However, there is a recent concern that statins may have an adverse effect on glucose metabolism [4]. Thus, re-evaluation of the comprehensive systemic benefits of LDL-lowering therapy in DM requires careful consideration and is of clinical importance [1]. Ezetimibe has been shown to reduce LDL [2] and ameliorate postprandial dyslipidemia [5] without adversely affecting glucose metabolism [1,6,7]. This suggests its potential appropriate usefulness for improving cardiovascular function and in pathological conditions in DM [8,9].

We hypothesized that ezetimibe monotherapy, as compared with atorvastatin monotherapy, as the LDL-lowering therapy could significantly improve microvascular endothelial dysfunction in patients with DM. To test this hypothesis, we prospectively enrolled stable patients with type 2 DM and mildly elevated LDL. Participants were randomly assigned to either ezetimibe or atorvastatin monotherapy and were studied for 6 months for the effects of the treatments on peripheral microvascular endothelial function assessed by RH-PAT examination.

## Methods

### Study population and study protocol

This was a single center, prospective, open-label, randomized, two-arm clinical trial to investigate the inter- and intra-group differences of the two strategies for LDL-lowering therapy on peripheral microvascular endothelial function in stable patients with type 2 DM. We primarily recruited lipid-lowering medication naïve patients with type 2 DM and mildly elevated LDL (LDL >120 mg/dL) through the outpatient clinic at Jinnouchi Hospital in Japan. We excluded patients with unstable conditions, coronary artery disease, stroke, cancer, active inflammation, autoimmune disease, lung disease, severe liver disease, and end-stage renal dysfunction. Patients were randomly allocated by using permuted-block randomization of six to either the ezetimibe group (10 mg/day) as the cholesterol absorption inhibition therapy or the atorvastatin group (5 mg/day) as the cholesterol synthesis suppression therapy for 6 months. We repeatedly measured serum lipid concentrations and peripheral microvascular endothelial function before and after each therapy at the outpatient clinic at Jinnouchi Hospital. We did not change the anti-diabetic medication during the study period.

This study was conducted in accordance with the principles contained in the Declaration of Helsinki. The study protocol was approved by the Human Ethics Review Committee of Jinnouchi Hospital. Signed consent was obtained from each participant.

### Assessment of peripheral microvascular endothelial function

The principles and details of RH-PAT examination have been described previously [10,11]. Briefly, we volumetrically evaluated peripheral microvascular endothelial function in the fingertip by RH-PAT using the Endo-PAT2000 device (Itamar Medical, Israel). Measurements were taken when patients were in a stable condition and in the fasting state in the early morning before taking their medication. Patients were examined on a bed in the supine position after at least 10 min of rest in a temperature- and light-controlled environment. A blood pressure cuff was placed on the upper arm to be studied, while the contra-lateral arm served as a control. PAT probes were placed on one finger of each hand. After an equilibration period, baseline pulse volume amplitude was measured for each fingertip for 5 min. The cuff was inflated to 60 mmHg above systolic pressure or 200 mmHg for 5 min before being deflated to induce reactive hyperemia. After cuff deflation, PAT recordings were made for 10 min and RH-PAT data were automatically analyzed in real time using computer software (Endo-PAT2000 software, version 3.0.4). The RH-PAT index (RHI) reflects the extent of reactive hyperemia and was calculated as the ratio of the average amplitude of the PAT signal over 1 min starting 1.5 min after cuff deflation (control arm, A; occluded arm, C) divided by the average amplitude of the PAT signal in the 2.5 min before cuff inflation (baseline: control arm, B; occluded arm, D) according to the equation (C/D)/(A/B) [10]. We calculated the natural logarithmic transformation (Ln) of the RHI values, the LnRHI [12]. We were able to minimize intra- and inter-observer variability as RH-PAT measurements were analyzed with a computerized and automated algorithm in an operator-independent manner [13]. Previous studies have demonstrated that RH-PAT technology has excellent reproducibility [13,14].

### Blood tests

Fasting blood samples were collected from the antecubital vein in the morning before the therapies and after the 6 months of treatment with ezetimibe or atorvastatin. Blood analyses were conducted at the hospital laboratory for the measurement of blood glucose, hemoglobin A1c (HbA1c), cholesterol, and triglyceride. Blood lathosterol, campesterol, and non-esterified free fatty acid (NEFA) were measured by SRL Corp., Tokyo, Japan. Serum total cholesterol, triglyceride and NEFA were measured by enzymatic methods, and high-density lipoprotein (HDL) and LDL were measured by direct methods using commercial reagents (Sekisui Medical Co. Ltd., Tokyo). Fasting plasma glucose (FPG) was measured by the hexokinase method and HbA1c was measured by the latex agglutination immunoassay. Campesterol and lathosterol were determined by gas chromatography.

### Statistical analysis

Based on our preliminary examination at our hospital, power analysis indicated that an enrollment of 30 patients was required to detect a mean difference in percent change in LnRHI 20 ± 12% in the ezetimibe group and 10 ± 6% in the atorvastatin group, with a power of 80% and a two-sided alpha of 0.05. The results of the normally distributed continuous variables (determined by the Shapiro–Wilk test) are expressed as mean (standard deviation), while those of the continuous variables with skewed distributions are expressed as median values (interquartile range). Differences in baseline characteristics of the two groups were analyzed using the Student’s *t*-test, the Mann–Whitney U test or the Fisher’s exact test for categorical data, as appropriate. Either the paired Student’s *t*-test or the Wilcoxon test was used to analyze the effect of each treatment in intra-group analysis. The percent changes in LnRHI between the ezetimibe group and the atorvastatin group were further evaluated by analysis of variance (ANOVA) with adjustment for age, gender, and body mass index (BMI). Logistic regression analysis was used to evaluate the association between improvement in endothelial function (LnRHI increase >40%, the highest tertile) and baseline clinical variables, including age, gender, BMI, HbA1c, FPG, lipid parameters, medications, and LDL-lowering therapy allocation (ezetimibe or atorvastatin). Associations between groups and all other parameters were analyzed first by univariate logistic regression analysis, followed by multivariate logistic regression analysis using the forced inclusion model, and the Hosmer–Lemeshow goodness-of-fit statistic was calculated. To determine the relationship between changes in various clinical parameters and percent changes in LnRHI, correlations between variables of interest were analyzed using Pearson’s correlation coefficient. A p-value <0.05 was considered statistically significant. Statistical analyses were performed using the Statistical Package for Social Sciences, version 19 (SPSS Inc., IBM, Tokyo, Japan) and SAS, version 9.4 (SAS Institute Inc., Cary, NC, USA).

## Results

### Baseline clinical characteristics

The study comprised 33 stable Japanese patients with type 2 DM and mildly elevated LDL (LDL: 142 ± 21.7 mg/dL). Clinical baseline characteristics of patients and each group are shown in Table 1. Baseline characteristics of patients in the ezetimibe group were similar to those of the atorvastatin group. Baseline levels of BMI, HbA1c, FPG, total cholesterol, LDL, HDL, triglyceride, and NEFA were not significantly different between groups. There was no significant difference in peripheral microvascular endothelial function assessed by LnRHI values in the RH-PAT examination between groups at baseline (Table 2).

**Table 1:** **Baseline clinical characteristics**

	**Ezetimibe (** ***n*** **= 17)**	**Atorvastatin (** ***n*** **= 16)**	**p-value**
Age (years)	64.2 ± 9.8	65.0 ± 8.0	0.81
Sex, male (%)	7 (41.2%)	8 (50.0%)	0.73
Body mass index (kg/m^2^)	22.5 ± 2.4	23.3 ± 3.5	0.45
Hypertension (%)	9 (56.3%)	9 (52.9%)	1.00
Current smoking (%)	4 (23.5%)	4 (25.0%)	1.00
Hemoglobin A1c (%)	7.3 ± 1.1	6.9 ± 0.6	0.18
Fasting plasma glucose (mg/dL)	135.8 ± 21.5	131.4 ± 21.4	0.57
Duration of diabetes (yeas)	13.6 ± 8.1	11.9 ± 9.3	0.58
Total cholesterol (mg/dL)	237.5 ± 25.8	226.6 ± 29.5	0.27
LDL cholesterol (ng/mL)	148.8 ± 22.1	135.6 ± 19.7	0.08
HDL cholesterol (mg/dL)	57.0 ± 11.3	54.9 ± 19.0	0.71
Triglyceride (mg/dL)	112.0 (100.0–192.5)	134.5 (76.3–229.3)	0.83
Anti-diabetic medicines	-	-	-
Sulfonylureas (%)	2 (11.8%)	5 (31.5%)	0.23
Metformin (%)	5 (29.4%)	4 (25.0%)	1.00
Alpha-glucosidase inhibitor (%)	5 (29.4%)	6 (37.5%)	0.72
Thiazolidinedione (%)	1 (5.9%)	2 (12.5%)	0.60
Glinide (%)	0 (0%)	1 (6.3%)	0.49
DPP-4 inhibitor (%)	1 (5.9%)	2 (12.5%)	0.60
Insulin (%)	8 (47.1%)	3 (18.8%)	0.14
Any anti-diabetic medications (%)	14 (82.3%)	14 (87.5%)	0.53
LnRHI	0.471 ± 0.157	0.552 ± 0.084	0.076

LDL: low-density lipoprotein, HDL: high-density lipoprotein, DPP-4: dipeptidyl peptidase-4, LnRHI: natural logarithmic transformed value of reactive hyperemia peripheral tonometry index.

**Table 2:** **Changes in diabetes and lipid parameters and LnRHI**

	**Ezetimibe (** ***n*** **= 17)**			**p − value**	**Atorvastatin (** ***n*** **= 16)**			**p − value**
	**Baseline**		**6 months**		**Baseline**		**6 months**	
Hemoglobin A1c (%)	7.3 ± 1.1		7.5 ± 0.8	0.307	6.9 ± 0.6		6.7 ± 0.7	0.062
% change (%)		3.9 ± 13.3				−0.3 ± 2.1		
Fasting plasma glucose (mg/dL)	135.8 ± 21.5		135.8 ± 27.9	0.581	131.4 ± 21.4		133.9 ± 25.5	0.651
% change (%)		2.6 ± 15.3				2.8 ± 7.8		
Total cholesterol (mg/dL)	237.5 ± 25.8		195.7 ± 21.6	<0.001	226.6 ± 29.5		174.4 ± 22.2	<0.001
% change (%)		−17.1 ± 9.3‡				−22.8 ± 6.1‡		
LDL cholesterol (mg/dL)	148.8 ± 22.1		115.5 ± 17.9	<0.001	135.6 ± 19.7		88.9 ± 17.5	<0.001
% change (%)		−21.9 ± 9.6*				−34.5 ± 7.8*		
HDL cholesterol (mg/dL)	57.0 ± 11.3		54.9 ± 12.6	0.31	54.9 ± 19.0		58.0 ± 18.5	0.035
% change (%)		−3.4 ± 12.1‡				6.5 ± 10.5‡		
Triglyceride (mg/dL)	112.0 (100.0–192.5)		94.0 (80.0–171.0)	0.18	134.5 (76.3–229.3)		105 (67.0–188.3)	0.12
% change (%)		−8.9 ± 28.1				−7.0 ± 45.0		
NEFA (mg/dL)	561.1 ± 236.8		429.7 ± 195.9	0.009	538.8 ± 319.5		520.2 ± 227.3	0.746
% change (%)		−19.9 ± 27.4‡				11.3 ± 44.1‡		
Lathosterol (μg/mL)	3.30 ± 1.45		4.60 ± 1.96	0.004	3.44 ± 1.52		1.57 ± 0.61	<0.001
% change (%)		51.2 ± 52.2*				−42.0 ± 49.7*		
Campesterol (μg/mL)	6.14 ± 2.74		2.69 ± 0.72	<0.001	6.56 ± 1.53		6.21 ± 1.86	0.217
% change (%)		−51.0 ± 17.7*				−6.5 ± 23.3*		
Lathosterol/campesterol	0.457 (0.337–1.006)		1.428 (1.189–2.303)	<0.001	0.593 (0.282–0.678)		0.276 (0.205–0.311)	0.002
% change (%)		239.3 ± 38.5*				−39.2 ± 38.5*		
LnRHI	0.471 ± 0.157		0.678 ± 0.187	0.007	0.552 ± 0.084		0.558 ± 0.202	0.645
% change (%)		63.3 ± 89.2‡				7.4 ± 41.2‡		

LDL: low-density lipoprotein, HDL: high-density lipoprotein, NEFA: non-esterified fatty acid, LnRHI: natural logarithmic transformed value of reactive hyperemia peripheral tonometry index. * p < 0.001, ‡ p < 0.05; ezetimibe *vs*. atorvastatin.

### Changes in HbA1c, FPG, and blood lipid parameters

**Figure 1:**
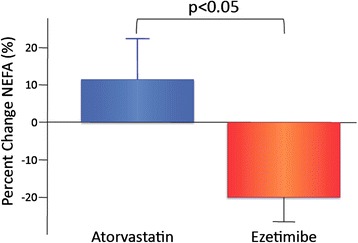
Percent changes in NEFA with atorvastatin and ezetimibe therapies. Percent changes in NEFA = (post-NEFA - pre-NEFA) × 100 / pre-NEFA. Bar graphs represent data of mean and standard error of mean. Atorvastatin group (*n* = 16) and ezetimibe group (*n* = 17). NEFA: non-esterified free fatty acid.

**Figure 2:**
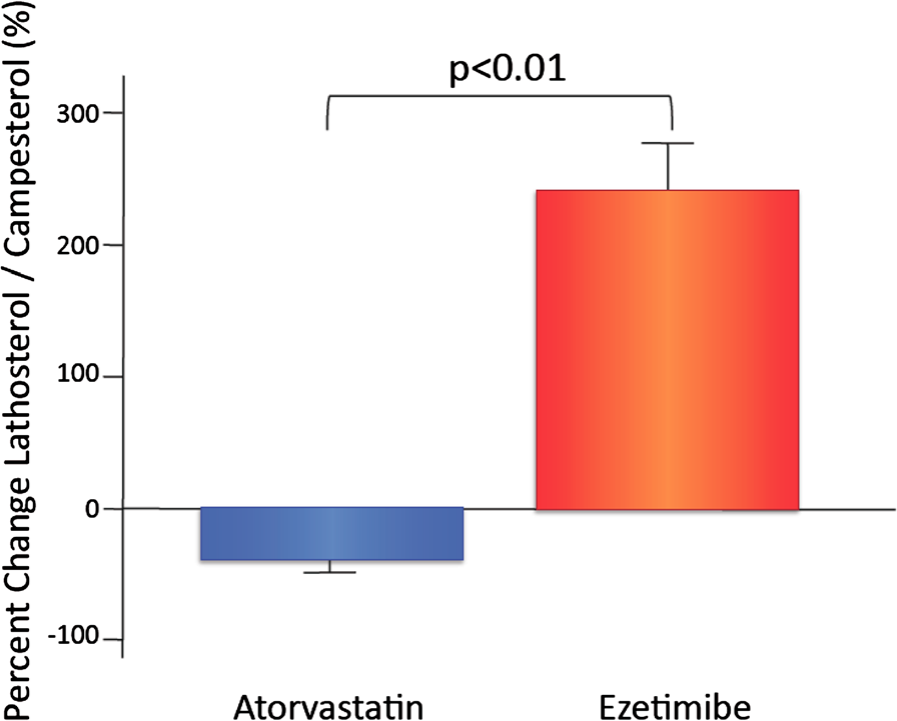
Percent changes in lathosterol/campesterol with atorvastatin and ezetimibe therapies. Percent changes in lathosterol/campesterol = (post-lathosterol/campesterol - pre-lathosterol/campesterol) × 100 / pre-lathosterol/campesterol. Bar graphs represent data of mean and standard error of mean. Atorvastatin group (*n* = 16) and ezetimibe group (*n* = 17).

All patients presented with non-significant changes in HbA1c (7.1 ± 0.9% to 7.1 ± 0.9%, p = 0.98) and FPG (133.7 ± 21.2 to 136.4 ± 26.4 mg/dL, p = 0.47). Intra-group changes in HbA1c and FPG were not statistically significant in both groups during therapies. As shown in Table 2, total cholesterol and LDL significantly decreased in both groups, but decreases in total cholesterol and LDL were significantly greater in the atorvastatin group than in the ezetimibe group (total cholesterol: −22.8 ± 6.1% *vs.* −17.1 ± 9.3%, p < 0.05; LDL: −34.5 ± 7.8% *vs.* −21.9 ± 9.6%, p < 0.01). NEFA significantly decreased in the ezetimibe group but not in the atorvastatin group (Table 2). The percent decrease in NEFA was significantly greater in the ezetimibe group compared with the atorvastatin group (−19.9 ± 27.4% *vs.* 11.3 ± 44.1%, p < 0.05; Table 2 and Figure 1). Campesterol, as the cholesterol absorption marker, significantly decreased in the ezetimibe group but not in the atorvastatin group. Lathosterol, as the cholesterol synthesis marker, significantly decreased in the atorvastatin group and increased in the ezetimibe group. The lathosterol to campesterol ratio, as the comprehensive indicator of cholesterol synthesis and absorption balance, exhibited a significant increase in the ezetimibe group and a significant decrease in the atorvastatin group. The percent change in the lathosterol to campesterol ratio was significantly different between the two groups (ezetimibe group: 239.3 ± 38.5%, atorvastatin-group: −39.2 ± 38.5%, p < 0.001; Table 2 and Figure 2).

### Changes in peripheral microvascular endothelial function assessed by LnRHI

**Figure 3:**
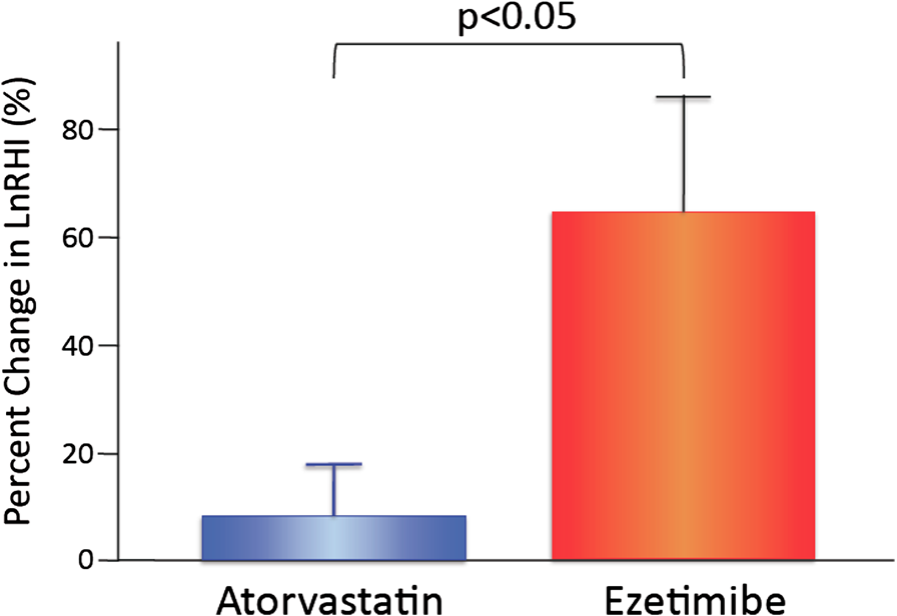
Percent changes in LnRHI with atorvastatin and ezetimibe therapies. Percent changes in LnRHI = (post-LnRHI - pre-LnRHI) × 100 / pre-LnRHI.Bar graphs represent data of mean and standard error of mean. Atorvastatin group (*n* = 16) and ezetimibe group (*n* = 17). LnRHI: natural logarithmic transformed value of reactive hyperemia peripheral arterial tonometry index.

During the 6-month treatments, microvascular endothelial function assessed by LnRHI significantly improved after LDL-lowering therapy in all patients (LnRHI: 0.510 ± 0.131 to 0.630 ± 0.198, p = 0.01). Ezetimibe monotherapy but not atorvastatin monotherapy exhibited a significant intra-group improvement in peripheral microvascular endothelial function assessed by LnRHI (ezetimibe group: 0.471 ± 0.157 to 0.678 ± 0.187, p < 0.01; atorvastatin group: 0.552 ± 0.084 to 0.558 ± 0.202, p = 0.64; Table 2). Regarding inter-group comparisons, the percent changes in LnRHI were significantly greater in the ezetimibe group compared with the atorvastatin group (p = 0.028; Table 2 and Figure 3). After using ANOVA with adjustment for age, gender, and BMI, the percent changes in LnRHI were significantly greater in the ezetimibe group compared with the atorvastatin group (ANOVA, p = 0.041).

Univariate logistic regression analysis for various clinical factors demonstrated that only ezetimibe therapy significantly correlated with improvements in peripheral microvascular endothelial function defined as the highest tertile of the percent changes in LnRHI (Table 3, odds ratio: 4.88, 95% confidence interval: 1.01–23.57, p = 0.049). Forced inclusion multivariate logistic regression analysis with age, gender, and the cholesterol-lowering therapy allocation revealed that ezetimibe therapy significantly correlated with improvements in endothelial function (Table 3, odds ratio: 6.42, 95% confidence interval: 1.13–36.47, p = 0.036). The Hosmer–Lemeshow statistic was appropriate (p = 0.53).

**Table 3:** **Logistic regression analysis of baseline parameters for the improvement of LnRHI (>40%)**

**Baseline variable**	**Univariate regression**			**Multivariate regression using forced inclusion model**		
	**OR**	**95% CI**	**p**	**OR**	**95% CI**	**p**
Age (per year)	1.02	0.94–1.11	0.58	1.05	0.95–1.15	0.33
Gender (male)	2.28	0.54–9.67	0.27	4.22	0.71–25.03	0.11
Body mass index (per 1.0)	1.06	0.83–1.36	0.66	-	-	-
Hemoglobin A1c (per 0.1; %)	0.98	0.89–1.04	0.33	-	-	-
Fasting plasma glucose (per 1.0; mg/dl)	0.98	0.94–1.01	0.17	-	-	-
Total cholesterol (per 1.0; mg/dl)	0.99	0.97–1.03	0.96	-	-	-
LDL cholesterol (per 1.0; mg/dl)	0.98	0.95–1.01	0.21	-	-	-
HDL cholesterol (per 1.0; mg/dl)	1.01	0.96–1.06	0.72	-	-	-
Triglyceride (per 1.0; mg/dl)	1.01	0.99–1.02	0.20	-	-	-
Sulfonylureas (yes)	1.42	0.26–7.76	0.69	-	-	-
Metformin (yes)	1.60	0.34–7.65	0.56	-	-	-
Alpha-glucosidase inhibitor (yes)	0.54	0.11–2.62	0.45	-	-	-
Thiazolidinedione (yes)	0.86	0.07–1.66	0.91	-	-	-
Glinide (yes)	1.00	0.00–---	1.00	-	-	-
DPP4 inhibitor (yes)	0.86	0.07–10.66	0.91	-	-	-
Insulin (yes)	1.74	0.40–7.91	0.45	-	-	-
Ezetimibe-therapy (yes)	4.88	1.01–23.57	0.049	6.42	1.13–36.47	0.036

LnRHI: natural logarithmic transformed value of reactive hyperemia peripheral tonometry index; OR, odds ratio; CI, confidence interval; LDL, low-density lipoprotein; HDL, high-density lipoprotein, DPP-4, dipeptidyl peptidase-4; Hosmer–Lemeshow p = 0.53 in multivariate analysis.

### Correlation between percent changes in LnRHI and various lipid parameters

To determine the associated factors between the LDL-lowering therapy-induced improvement in microvascular endothelial function and changes in lipid parameters, we investigated the correlation coefficient between percent changes in LnRHI and percent changes in plasma lipid variables during therapy. As shown in Table 4, percent changes in LnRHI did not significantly correlate with percent changes in total cholesterol, LDL, HDL, and triglyceride. Changes in LnRHI showed significant correlations with changes in cholesterol absorption or synthesis parameters during therapy (Table 4). The indicator of cholesterol synthesis and absorption balance (the ratio of lathosterol to campesterol) demonstrated the greatest correlation efficient value (r = 0.459, p = 0.008) among all parameters. In the intra-group analyses, none of the parameters were significantly correlated with percent changes in LnRHI in the ezetimibe and atorvastatin groups (Tables 5 and 6).

**Table 4:** **Correlation between percent changes in lipid parameters and percent changes in LnRHI in all subjects**

	**r**	**p-value**
Percent change in total cholesterol	−0.053	0.769
Percent change in LDL cholesterol	−0.053	0.770
Percent change in HDL cholesterol	−0.222	0.215
Percent change in triglyceride	0.236	0.186
Percent change in NEFA	−0.115	0.524
Percent change in lathosterol	0.354	0.047
Percent change in campesterol	−0.399	0.024
Percent change in lathosterol/campesterol	0.459	0.008

LDL: low-density lipoprotein, HDL: high-density lipoprotein, NEFA: non-esterified fatty acid, LnRHI: natural logarithmic transformed value of reactive hyperemia peripheral tonometry index.

**Table 5:** **Correlation between percent changes in lipid parameters and in LnRHI in subjects treated by ezetimibe**

	**r**	**p-value**
Percent change in total cholesterol	−0.246	0.336
Percent change in LDL cholesterol	−0.375	0.138
Percent change in HDL cholesterol	−0.220	0.397
Percent change in triglyceride	0.150	0.565
Percent change in NEFA	0.137	0.601
Percent change in lathosterol	0.283	0.289
Percent change in campesterol	−0.273	0.306
Percent change in lathosterol/campesterol	0.324	0.221

LDL: low-density lipoprotein, HDL: high-density lipoprotein, NEFA: non-esterified fatty acid, LnRHI: natural logarithmic transformed value of reactive hyperemia peripheral tonometry index.

**Table 6:** **Correlation between percent changes in lipid parameters and in LnRHI in subjects treated by atorvastatin**

	**r**	**p-value**
Percent change in total cholesterol	−0.106	0.696
Percent change in LDL cholesterol	−0.420	0.105
Percent change in HDL cholesterol	0.276	0.300
Percent change in triglyceride	0.438	0.065
Percent change in NEFA	−0.055	0.893
Percent change in lathosterol	−0.180	0.504
Percent change in campesterol	−0.098	0.723
Percent change in lathosterol/campesterol	−0.121	0.655

LDL: low-density lipoprotein, HDL: high-density lipoprotein, NEFA: non-esterified fatty acid, LnRHI: natural logarithmic transformed value of reactive hyperemia peripheral tonometry index.

## Discussion

This is the first study to directly investigate the effectiveness of two different LDL-lowering strategies, cholesterol absorption inhibition and cholesterol synthesis suppression, on microvascular endothelial function in type 2 DM. We observed that ezetimibe monotherapy, when compared with atorvastatin monotherapy, significantly decreased serum NEFA levels and significantly improved peripheral microvascular endothelial function using RH-PAT tests in stable patients with type 2 DM.

In patients with type 2 DM, lowering cholesterol using statins is clinically effective and is an established treatment for preventing cardiovascular events [3]. However, there are no clinical studies comparing the effects of LDL-lowering monotherapies, such as cholesterol synthesis suppression using statins or cholesterol absorption inhibition using ezetimibe, on endothelial function in patients with type 2 DM.

In the small intestine, cholesterol is absorbed through NPC1L1, the target molecule of ezetimibe [2]. Ezetimibe exhibits its LDL-lowering properties by blocking NPC1L1 in the small intestine without attenuating the mevalonate pathway [2]. Recently, it has been reported that inactivating mutations in the NPC1L1 gene provided significant protection against coronary heart disease [15]. It has also been reported that intestinal expression of NPC1L1 increased in patients with type 2 DM [16]. Additionally, the LDL-lowering effect of ezetimibe was significantly greater in DM [17], suggesting the potentially beneficial role of ezetimibe as a NPC1L1 inhibitor in the treatment of DM with dyslipidemia and vascular dysfunction [8,9].

In the present study, each monotherapy resulted in a significant decrease in blood LDL, but the marker of the cholesterol synthesis/absorption balance showed a markedly different effect. We confirmed that the two treatments had opposite effects on endogenous metabolism of cholesterol synthesis/absorption, and found that changes in the cholesterol synthesis/absorption balance significantly correlated with changes in microvascular endothelial function in patients with DM. Interestingly, the effect of each monotherapy on peripheral microvascular endothelial function in patients with DM varied significantly. Ezetimibe monotherapy but not atorvastatin monotherapy significantly improved peripheral microvascular endothelial function. Effective cardiovascular actions through the inhibition of cholesterol absorption by ezetimibe would be clinically expected in DM [9].

Previously, Nochioka *et al.* reported that ezetimibe, when compared with pravastatin, significantly improved endothelial function as assessed by flow-mediated dilation (FMD) in young healthy volunteers [18]. The researchers identified a significant correlation between the reduction of a cholesterol absorption marker and improvement in FMD. In outpatients with type 2 DM, we found similar clinical results in the present study. Recently, the effect of pravastatin or ezetimibe on endothelial function was reported as evaluated by FMD in non-DM patients with hypercholesterolemia [19]. The researchers concluded that LDL-lowering by pravastatin or ezetimibe induced similar effects on vascular endothelial function improvement, and they suggested that the pleiotropic effects of statins were not important for improvement in vascular endothelial function. Although the LDL decrease was equal in the two treatment strategies in that study, the improvement in FMD tended to be greater with ezetimibe than with pravastatin (FMD change: ezetimibe 5.2% *vs.* pravastatin 3.8%), indicating the potentially beneficial use of ezetimibe. The major differences between that study and the present study are the baseline clinical backgrounds of patients (non-DM *vs.* DM and hypercholesterolemia [LDL: 180 mg/dL] *vs.* mildly elevated cholesterol [LDL: 142 mg/dL]), treatment period (6 weeks *vs.* 6 months), and the method of assessing endothelial function (FMD *vs.* RH-PAT). Several previous studies failed to demonstrate the beneficial effect of ezetimibe monotherapy on endothelial function [20-22]. These studies compared the effects of statin or ezetimibe on endothelial function in patients with heart failure [20,21] or coronary artery disease with a small number of patients with DM [22], and the authors mentioned the ‘pleiotropic effect of statin’ for the treatment of cardiovascular disease. In contrast, Yunoki et al. reported the improvement achieved by ezetimibe on endothelial dysfunction in the postprandial phase [23] and in CAD patients with hypertriglyceridemia [24]. Moreover, Yamaoka-Tojo et al. reported the benefit of the add-on therapy of ezetimibe to statin therapy in patients at high risk of cardio-metabolic disease [25].

In the present study, atorvastatin did not significantly improve microvascular endothelial function in DM, although there was a greater reduction in LDL. The reason why atorvastatin did not improve endothelial function is unclear. Several clinical studies reported that statins could improve endothelial function in patients with DM [26-28]. In contrast, several randomized trials showed that statins did not improve endothelial function in patients with type 2 DM [29-32]. Recently, Zhang et al. reported a meta-analysis of placebo randomized studies using FMD as the measure of endothelial function to determine the effects of statins on endothelial function in patients with DM [33]. They found overall beneficial effects of statins on endothelial function in patients with DM but the beneficial effects were not observed in patients with type 2 DM, obesity (BMI > 27), and older age (>55 years) in the sub-analysis. In the present study, all of the enrolled patients had type 2 DM and average age of 64.6 years. Thus, we speculated that our patients could be less responsive to statin therapy in terms of endothelial function improvement.

In the present study, we investigated the effects of ezetimibe and atorvastatin on the peripheral microvascular endothelial function assessed by RH-PAT. We focused mainly on the microcirculation rather than the large conduit arteries, such as the brachial artery that was used for the FMD measurement. Regarding the effect of statins on microvascular endothelial function, previous studies reported that there was no significant benefit of statin therapy in patients with DM [34-37]. This study is the first to report the improvement of microvascular endothelial dysfunction by ezetimibe in patients with type 2 DM.

Because LDL-lowering therapy is essential and effective for patients with DM in the clinical practice, we cannot conduct a placebo-controlled study with statin therapy for DM. We think that the results of the present study will shed light on the controversial subject of endothelial dysfunction therapy in patients with DM. Further studies evaluating larger numbers of DM cases are required to confirm the results of the present study.

The clinical properties of ezetimibe for the treatment of dyslipidemia are improvements in both hypercholesterolemia and postprandial dyslipidemia [2,38]. In the present study, we identified, for the first time, that ezetimibe significantly reduced NEFA in patients with DM. It has been reported that ezetimibe can significantly reduce fatty acid absorption in animal models [39], that NEFA is involved in the pathological conditions of insulin resistance [40], and that NEFA is associated with inflammation [41,42] and endothelial dysfunction [43]. These findings suggest that NEFA could have great significance in the pathological conditions of DM [44]. In the present study, ezetimibe significantly reduced NEFA in patients with DM, but statin did not have the similar effect. Changes in NEFA might bring about different effects on the long-term metabolic balance of lipids and glucose [43]. A potential therapy to significantly reduce NEFA in DM has not been reported until now, and we think that this result has important clinical meaning. The impact of ezetimibe-mediated changes in NEFA on vascular endothelial function requires further detailed examination.

Both ezetimibe and atorvastatin therapy resulted in significant reductions of LDL; however, ezetimibe significantly improved endothelial function compared to atorvastatin. This finding suggests that ezetimibe added on to the LDL-lowering effects by an unknown mechanism, and this unique property might contribute to the improvement of endothelial function in patients with DM. It has been reported that a postprandial metabolic disorder is deeply involved in endothelial dysfunction in DM, and ezetimibe improved postprandial hyperlipemia and its induced endothelial dysfunction [23]. We did not measure postprandial lipid parameters in the present study, but we can speculate that the ezetimibe-mediated improvement in postprandial dyslipidemia may potentially contribute to the beneficial vascular function. In a future study, we need to investigate the correlation between ezetimibe-mediated improvement of postprandial dyslipidemia and endothelial function in patients with DM.

Recent meta-analysis demonstrated that the statin-mediated increase in HDL might not be significantly associated with the decrease in future cardiovascular events [45]. Regarding HDL, it has been reported that not only plasma quantity of HDL, but also functional quality of HDL is important for exerting vascular protective and anti-atherosclerotic effects [46]. In the present study, the HDL increase by atorvastatin was not associated with improvement of endothelial function in patients with DM. It will be worth to determine whether ezetimibe could modulate HDL function in a future study. Based on the present results, we think that changes in HDL by LDL-lowering therapy cannot be good indicators of improvement of microvascular endothelial function in patients with DM.

This study had several limitations. The study was a single center study and the protocol was open-labeled. Only a small number of patients made up the study population, and the study period was short. We did not evaluate serum levels of postprandial lipids and oxysterol. As the study patients were stable and had relatively favorable and controlled type 2 DM, were not obese, and did not have cardiovascular diseases, further studies are required using a larger number of patients with DM with a broad spectrum of clinical backgrounds.

In conclusion, both ezetimibe monotherapy and atorvastatin monotherapy significantly reduced LDL, and ezetimibe monotherapy significantly increased LnRHI in patients with DM. Ezetimibe, but not atorvastatin, significantly reduced serum levels of NEFA. In this population of patients with type 2 DM, ezetimibe monotherapy significantly ameliorated dyslipidemia and improved peripheral microvascular endothelial dysfunction.

## Abbreviations

BMI: Body mass index
DM: Diabetes mellitus
FPG: Fasting plasma glucose
FMD: Flow-mediated dilation
HbA1c: Hemoglobin A1c
HDL: High-density lipoprotein cholesterol
LDL: Low-density lipoprotein cholesterol
RH-PAT: Reactive hyperemia peripheral arterial tonometry
LnRHI: Natural logarithmic transformed value for the RH-PAT index
NPC1L1: Niemann-Pick C1-Like 1
NEFA: Non-esterified free fatty acid
RHI: RH-PAT index
